# Idiopathic Scrotal Calcinosis: A Case Report of a Rare Entity

**DOI:** 10.1155/2019/6501964

**Published:** 2019-12-16

**Authors:** Umesh Jayarajah, Lalani de Silva, Chandu de Silva, Sanjeewa Seneviratne

**Affiliations:** ^1^Department of Surgery, Faculty of Medicine, University of Colombo, Colombo, Sri Lanka; ^2^Department of Pathology, Faculty of Medicine, University of Colombo, Colombo, Sri Lanka

## Abstract

Scrotal calcinosis is a benign condition where multiple calcified nodules are found within the dermis of the scrotal skin. It is a rare condition which is usually asymptomatic and has no clear aetiology although several theories have been proposed in the aetiopathogenesis. We report a 55-year-old man with extensive scrotal calcinosis. Surgical excision of the affected scrotal skin was curative.

## 1. Introduction

Idiopathic scrotal calcinosis is a benign condition involving the scrotal skin defined as presence of multiple calcified nodules confined to the dermis [1]. Although it is believed to be idiopathic, dystrophic calcification of longstanding sebaceous cysts, and degenerative changes of the dartos are postulated to be involved in the pathogenesis [1]. The knowledge regarding this condition is limited only to case reports and series due to the rarity of the disease. Currently, surgery remains the only curative option. We report a 55-year-old man presenting with this rare condition with a review of literature.

## 2. Case Presentation

**Figure 1:**
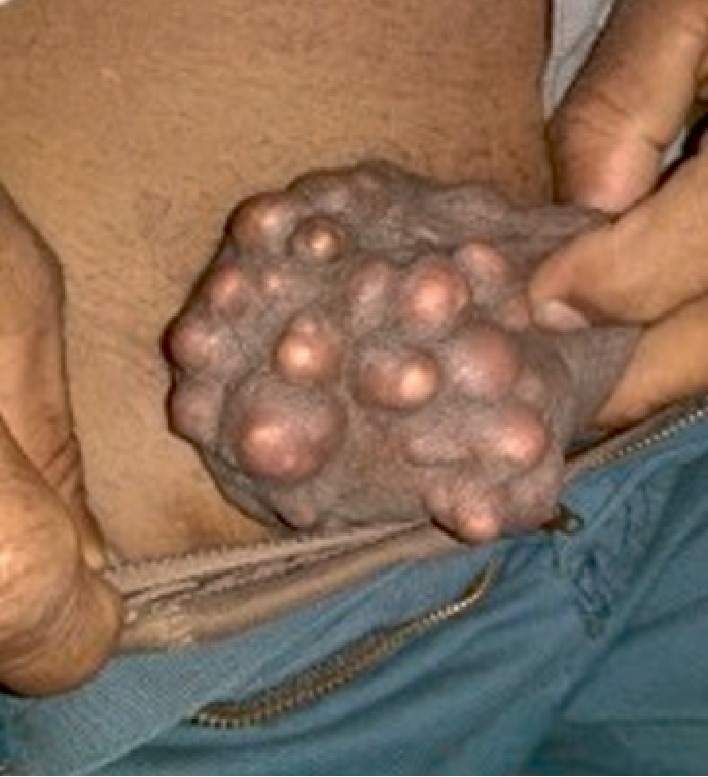
Physical examination showing multiple, firm, nontender, subcutaneous nodules studded within the scrotal wall.

A 55-year-old Sri Lankan Sinhalese manual labourer presented with progressively increasing multiple, painless nodules in the scrotum of 4 year duration. He did not have any other medical comorbidities and was otherwise asymptomatic. There was no history of discharge, ulcers, or infection in relation to the scrotal lesions. Physical examination showed multiple, firm, nontender, subcutaneous nodules studded within the scrotal wall (Figure 1). Serum calcium, phosphorous, and parathormone levels were within normal limits. He underwent excision of all the lesions through multiple skin stabs under spinal anaesthesia with a satisfactory cosmetic result.

**Figure 2:**
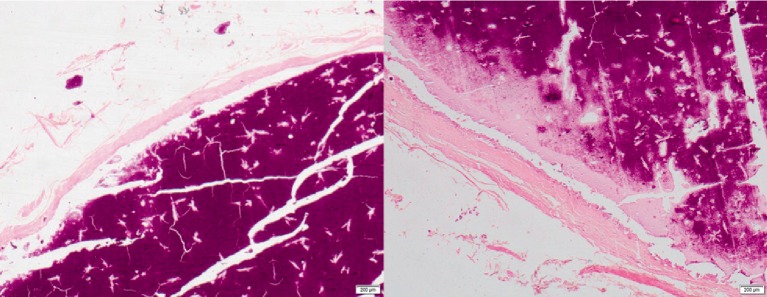
Histology showing calcified granular material surrounded by thin fibrotic wall (H & E ×200).

Pathological examination revealed forty three tan colour nodules, the sizes of which ranged from 23 mm to 5 mm. Microscopy revealed basophilic granular calcified material surrounded by thin fibrotic wall, devoid of an epithelial lining. Foreign body type chronic inflammatory reaction was noted in some areas. There was no evidence of granulomatous reaction (Figure 2). There was no evidence of recurrence at six month follow-up.

## 3. Discussion

Idiopathic scrotal calcinosis was first described by Lewinski in 1883 [2].The condition usually begins in adolescence or early adulthood. It usually present as yellowish brown, firm, solitary, or multiple nodular lesions on the scrotal skin which are increasing in size and number with time. Usually they are asymptomatic but may produce vague pain, discharge or itching and may also be complicated with infections [2]. Although it involves calcium deposition, there is no association with abnormal calcium metabolism [3]. Indication for surgery is usually cosmetic and in rare occasions, for symptom relief.

Although scrotal calcinosis is believed to be idiopathic in the majority, the pathogenesis is still controversial. Dystrophic calcification secondary to sebaceous cysts, eccrine epithelial cyst, or degenerated dartos muscle are postulated as possible mechanisms by several authors [3–5]. Calcification of sebaceous cysts may occur after an infection that later progresses to a degenerative process and results in the loss of epithelial lining of the cyst wall with calcification of the substance [4]. Ito et al. proposed an eccrine epithelial cyst origin with excessive discharge and collection of debris in the lumen as the possible mechanism following immunohistochemical analyses [5]. If the above features are absent in the histological evaluation similar to the presented case, the origin is considered idiopathic.

Currently, surgery remains the only recommended treatment which provides excellent cosmetic outcomes while enabling the pathological confirmation of diagnosis. As the disease is confined to the scrotal dermis, excision should be limited to the skin. Some authors have used partial scrotectomy and reconstruction in cases with extensive disease [1, 6]. There is lack of data on recurrence however, some authors propose a high incidence of recurrence in the long term [6]. Complete excision including the smallest lesion is believed to be key in minimising recurrence [1, 6].

## 4. Conclusion

Idiopathic scrotal calcinosis is a rare and benign condition with an unclear pathogenesis. In some cases, the disease is extensive requiring scrotal skin excision. We report a similar case with extensive idiopathic scrotal calcinosis which was excised through multiple stabs. Although the depth of the excision is limited to the dermis, complete excision of even the smallest of lesions is required to minimize recurrences.
